# Defending against deepfake speech attacks via feature dropout and dropin regularization in large audio language models

**DOI:** 10.3389/frai.2026.1928592

**Published:** 2026-10-06

**Authors:** Yupei Li, Yixiong Fang, Jiahao Xue, Manuel Milling, Björn W. Schuller

**Affiliations:** 1 Group on Language, Audio, & Music (GLAM), Imperial College London, London, United Kingdom; 2 Chair of Health Informatics (CHI), TUM University Hospital, Munich, Germany; 3 Shanghai Jiaotong University, Shanghai, China; 4 Queen Mary University of London, London, United Kingdom; 5 Munich Center for Machine Learning (MCML), München, Germany; 6 Munich Data Science Institute (MDSI), München, Germany

**Keywords:** artificial intelligence, audio, deepfake, dropout, LLM

## Abstract

Deepfakes have raised widespread concern owing to their threats to privacy, security, and societal trust, driving growing research interest in effective detection methods. Audio deepfake detection has moved from handcrafted-feature classifiers to end-to-end deep learning architectures and, more recently, to self-supervised speech foundation models and general-purpose speech recognizers such as Whisper. The use of instruction-following speech large language models (LLMs), of which Speech Audio Language Music Open Neural Network (SALMONN) is a representative example, remains comparatively less explored, and forms the setting of this study. We argue that large language models (LLMs), with their powerful representational and information retrieval capabilities, hold significant potential for this task—provided that relevant audio features are carefully selected and prioritized. To this end, we employ a speech LLM, specifically SALMONN, for deepfake audio detection, incorporating a two-stage training strategy in which Stage 1 injects acoustic-feature awareness into the Low-Rank Adaptation (LoRA) adapters via an auxiliary feature-prediction pretext task (referred to as feature dropin), and Stage 2 performs windowed random retention on the encoder token sequence (referred to as feature dropout) before the final real/fake decision. Our approach achieves an absolute accuracy improvement of 0.188 on the FakeOrReal benchmark and attains state-of-the-art performance on the Automatic Speaker Verification Spoofing and Countermeasures (ASVspoof) 2019 LA subset with an Equal Error Rate (EER) of 0.00496, representing a 33.0% relative improvement over prior methods. Furthermore, the model transfers non-trivially to an out-of-domain artificial intelligence (AI)-generated music dataset (M6), reaching accuracy comparable to a ResNet18 baseline reported in prior study, which we interpret as evidence of transferable audio representations rather than broad cross-domain robustness.

## 1 Introduction

The detection of deepfake audio and speech has become increasingly critical due to the potential social implications, particularly amid the rapid dissemination of misinformation on social media (Li M. et al., 2025). However, one of the key challenges for reliable detection approaches is the variety of strategies for generating deepfake audio. For instance, Voice Synthesis (VS) (Tan et al., 2021) is a common technique that combines real voice cloning with synthetic voices. Voice Conversion (VC) (Khanjani et al., 2021) modifies a speaker's voice to resemble another speaker's while preserving the original content. Furthermore, Text-to-Speech (TTS) models (Klein et al., 2024) convert written text into audio using audio generation models. Fundamentally, the challenge in detecting deepfake audio lies in the advanced capabilities of modern audio generation models, which can alter specific elements of the original audio while preserving other components, resulting in a high degree of similarity to authentic audio.

Related research has a long history and originates from efforts to address the challenges of spoof detection. Early approaches primarily relied on traditional ML models built on handcrafted acoustic features and simple classification methods. Commonly used acoustic features include Mel-Frequency Cepstral Coefficients (MFCCs) (Davis and Mermelstein, 1980), Silence Ratio (SR) (Rabiner and Juang, 1993), and Spectral Centroid (SC) (Tschacher et al., 2004), among others. These features could reveal subtle spectral artifacts introduced by synthetic voice generation (Sahidullah et al., 2015) or standard speech features (Hamza et al., 2022). Afterward, these features were typically fed into binary classification models such as Support Vector Machines (SVMs) (Cortes and Vapnik, 1995). However, these conventional methods are often insufficient for capturing the full complexity of high-quality audio recordings.

Deep learning techniques have increasingly dominated the field in recent years. Convolutional Neural Networks (CNNs), applied to image-like audio representations such as Mel-spectrograms, have been particularly effective. For example, ResNet (P et al., 2020) and SENet (Lai et al., 2019) have demonstrated strong performance in spoof detection. Given the sequential nature of audio signals, Recurrent Neural Networks (RNNs), such as deep bidirectional Long Short-Term Memory (DBiLSTM) networks, have also been widely adopted (Arif et al., 2021). Hybrid architectures further combine spatial and temporal modeling; for instance, CRNN-Spoof (Chintha et al., 2020) integrates CNN and RNN components to capture both dependencies in audio signals. More recent approaches incorporate attention mechanisms and are often transformer-based. Examples include ConvNext-Tiny with Whisper (Pham et al., 2024), the Self-Attention model with Hybrid Features (Huang and Pun, 2024), and the privacy-preserving model SafeEar (Li X. et al., 2024), all of which report improved spoof detection performance.

Large language models (LLMs) have recently demonstrated unprecedented capabilities across various deep learning applications. Their effectiveness stems from massive parameter counts and extensive training data, enabling performance beyond that of traditional single-task DL models. LLMs have shown strong abilities in information retrieval (Liu et al., 2024) and achieved high performance on downstream tasks such as classification (Huang and He, 2024). They have also proven effective in detecting AI-generated text (Prajapati et al., 2024), suggesting potential applicability to related detection tasks. Although originally designed for natural language processing (NLP), recent adaptations enable LLMs to process audio data, for example through Mel-spectrogram representations. One such model is the Speech Audio Language Music Open Neural Network (SALMONN) (Tang et al., 2023), which we explore in this study for audio deepfake detection. Although such fine-tuned or pretrained LLM detectors can be applied to deepfake detection, they do not explicitly emphasize which acoustic features matter and do not attempt to eliminate long-tail (Li et al., 2025b) and uninformative features from the encoder token sequence. Our novelty lies in the two-stage training strategy that emphasizes salient acoustic features.

Although deepfake detection is essentially a binary classification task (Li et al., 2024a) (Figure 1), it still faces common machine learning challenges such as overfitting. To improve model reliability, Fang et al. (2024) proposed a dropout-based decoding strategy for LLMs. This motivates us to explore dropout beyond its conventional use further. While dropout is widely applied at the network level to mitigate overfitting and improve efficiency (Yan and Shao, 2024), its use at the feature level has received relatively little attention. Islam and Glocker (2022) introduced a frequency-based randomized dropout method, though it has not yet been applied to speech tasks. To address this gap, we propose applying dropout at the feature level. By randomly omitting features, the model is encouraged to reduce reliance on individual features and improve generalization. As a mirroring technique for feature removal via dropout, we draw inspiration from the discussion on extending neural network strategies to add more layers during training (Smith et al., 2016). We further extend this concept by introducing a dropin strategy at the feature level, such as MFCC, SR, etc. Dropin enforces the model to prioritize specific features, thereby improving its audio comprehension before making a final decision. We evaluate our approach on the standard benchmark datasets (Reimao and Tzerpos, 2019) and the Automatic Speaker Verification Spoofing and Countermeasures (ASVSpoof) 2019 dataset (Wang et al., 2020), which aim to identify deepfake samples. Additionally, we evaluate the models on an entirely out-of-domain but conceptually related AI-generated music detection task.

**Figure 1:**
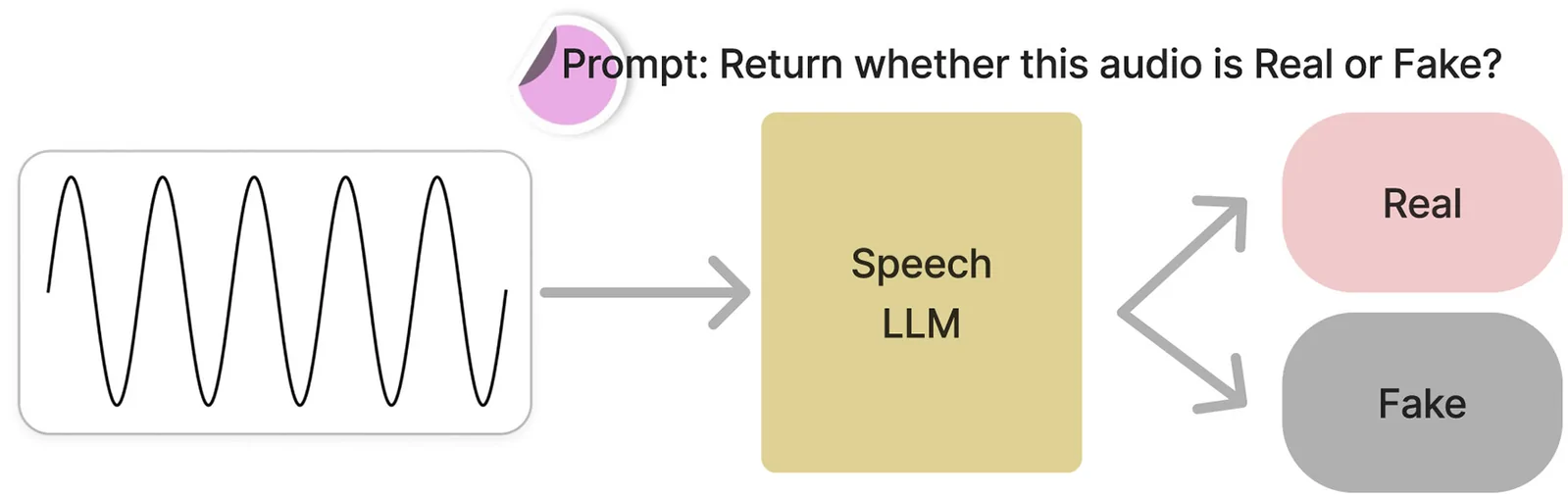
Basic LLMs pipeline for deepfake audio detection with changeable prompts.

Our contributions are twofold. First, we propose a two-stage adapter-training scheme for speech LLMs. Stage 1 (feature dropin) is an auxiliary supervision task in which the Low-Rank Adaptation (LoRA) adapters are trained to predict scalar acoustic descriptors (MFCC, Silence Ratio, and Spectral Centroid) from the audio, so that acoustic-feature awareness is 'dropped in' to the adapter weights. Stage 2 (feature dropout) applies windowed random retention over the SALMONN encoder token sequence during fine-tuning of the same adapters for real/fake classification. Note that the handcrafted feature values are never concatenated to the Stage 2 prompt; Stage 1 influences Stage 2 only through the shared LoRA parameters. Second, applied to the LLM SALMONN, our approach achieves state-of-the-art performance on in-domain deepfake speech detection and demonstrates strong out-of-domain generalization on AI-generated music detection.

## 2 Related study

### 2.1 Deepfake voice detection methods

Beyond conventional classifiers, deep learning has driven voice spoofing detection along four largely parallel lines, each of which we briefly characterize by its originality, method, and reported finding.

#### 2.1.1 Raw-waveform graph attention

End-to-End Spectro-Temporal Graph Attention Networks (RawGAT-ST) (Tak et al., 2021) was the first end-to-end system to combine a SincNet-style raw-waveform front-end with a heterogeneous spectro-temporal graph attention network, treating spectral and temporal sub-bands as graph nodes. On ASVspoof 2019 LA, it reports an Equal Error Rate (EER) of 1.06%, indicating that cross-band attention captures cues that CNN backbones miss, albeit at the cost of a specialized architecture without acoustic-feature priors. Audio Anti-Spoofing using Integrated Spectro-Temporal Graph Attention Networks (AASIST) (Jung et al., 2022) extends this line with heterogeneous stacking graph-attention layers and a maximal-graph operator that fuses spectral and temporal graphs, reaching 0.83% EER on the same benchmark; it improves generalization to unseen attacks but, like RawGAT-ST, provides no explicit account of which acoustic properties drive the decision.

#### 2.1.2 Self-supervised and pre-trained front-ends

Wav2Vec 2.0 (Baevski et al., 2020) and Emphasized Channel Attention, Propagation and Aggregation in Time Delay Neural Network (ECAPA-TDNN) (Desplanques et al., 2020) embeddings are widely reused as anti-spoofing front-ends: Wav2Vec fine-tuned with a lightweight classifier reaches sub-1 % EER on ASVspoof 2019 LA, and ECAPA-TDNN provides a compact speaker-verification-derived embedding. Both are strong feature extractors but rely on billion-scale pre-training and do not expose which acoustic properties the classifier ultimately uses. Explainable frameworks such as Channing et al. (2024) attempt to close this gap by attributing detection scores to time–frequency regions, yet the interpretability is *posthoc* and does not participate in training.

#### 2.1.3 Efficient and adaptive architectures

Nguyen Le et al. (2024) introduce a transformer-based few-shot detector that supports continuous adaptation to new attacks, addressing the practical need for online updates. Depthwise-Inception with contrastive training (Pham et al., 2025) reduces parameter count by an order of magnitude while remaining competitive under low-complexity budgets. These studies optimize the compute–accuracy trade-off but do not change the representation used for detection.

#### 2.1.4 Structured feature learning

SLIM (Zhu et al., 2024) exploits style–linguistics mismatch as a self-supervised signal, learning representations that separate speaker style from linguistic content and reporting strong cross-dataset generalization. RawBMamba (Chen et al., 2024) replaces attention with a bidirectional Mamba state-space model over raw audio, achieving competitive EER with near-linear time complexity. Both point to the value of structured priors, but the prior is baked into the architecture rather than into an interpretable supervision signal.

#### 2.1.5 Feature-engineering pipelines

A distinct line of very recent study returns to explicit acoustic feature engineering with modern selection tools. FakeSleuthNeXt (Celik and Akbal, 2026) combines a quantum-inspired local pattern generator over multi-level wavelet sub-bands with iterative feature selection (Iterative Neighborhood Component Analysis [INCA], IChi2, and IReliefF), and reports up to ~99% accuracy across five deepfake speech datasets. VoxVeritasNet (Celik et al., 2026b) advances this direction on the fake-or-real speech task by using a nine-level wavelet transform, a quantum-inspired dual-path mapping, and iterative selection, and does so with only about 20% of the training set—a setting directly comparable to ours. ETNeXt (Celik et al., 2026a), though targeting Brushless Direct Current (BLDC) motor-fault acoustic detection, exemplifies a principled multi-level wavelet + triadic-histogram + hybrid NCA/Chi-square feature pipeline that has been shown to transfer across acoustic domains.

#### 2.1.6 Demonstrated gap

Taken together, existing systems (i) either enlarge the front-end (Self-supervised learning [SSL] foundation models such as Wav2Vec 2.0, WavLM, and Cross-Lingual Speech Representation [XLS]-R) or the classifier (graph-attention back-ends such as AASIST and RawGAT-ST), (ii) rely on architectural priors (bidirectional Mamba, Depthwise-Inception) or expand feature banks through selection (FakeSleuthNeXt, VoxVeritasNet, and ETNeXt), and (iii) treat interpretability as *posthoc* attribution rather than as a training signal. None of them steers a speech-LLM through an *explicit acoustic-feature supervision signal* that participates in optimization, and none combines this signal with a controlled reduction of the encoder token sequence. This is the concrete gap our two-stage recipe addresses.

### 2.2 Dropin and dropout

Feature-level regularization for speech models has so far been dominated by four operators that differ in their axes of operation, rate structures, and locality. **Dropout** (Srivastava et al., 2014) applies an element-wise Bernoulli mask to neuron activations and rescales the survivors by 1/*p*; its variant, **Fast Dropout** (Wang and Manning, 2013), marginalizes the mask analytically while keeping the same axis. **DropConnect** (Wan et al., 2013) masks individual weights instead of activations, changing the axis but not the iid rate structure. **Maxout** (Goodfellow et al., 2013) does not remove units at all; it replaces activation pairs by their pointwise maximum, and any regularization is a by-product of aggregation rather than of information removal. **Frequency Dropout** (Islam and Glocker, 2022) is the closest prior operator: it is feature level. It drops entire frequency channels, but its axis is spectral channels with iid Bernoulli rates, and it has not been applied to speech-LLM encoder token sequences. Our feature dropout differs on all three axes simultaneously: it operates on the sequence axis of a speech-LLM's encoder output, uses a deterministic retention rate of 1/*w* per non-overlapping window with no rescaling, and preserves per-window locality so that no contiguous region is fully deleted.

For **Dropin**, prior study has interpreted the term as architectural growth—ExHuBERT (Amiriparian et al., 2024) block-extends HuBERT, and Large Language Model Meta AI Pro (LLaMA Pro) (Wu et al., 2024) grows LLaMA by block-wise weight duplication, framed by Li et al. (2025a) within a neuroplasticity-analog view. Our Stage-1 feature dropin is a different realization of the same intuition: no new layers or neurons are added; instead, we drop in *acoustic-feature awareness* into the existing LoRA adapters via an auxiliary regression pretext task on MFCC, Silence Ratio, and Spectral Centroid. To the best of our knowledge, feature-level dropin as pretext supervision—rather than as architectural growth—has not been investigated for speech-LLM anti-spoofing. Thus, contribution 1 (Stage-1 dropin) targets the fact that existing SSL front-ends and graph-attention back-ends do not expose which acoustic properties drive the decision, and contribution 2 (Stage-2 feature dropout) targets the fact that Dropout, DropConnect, Maxout, and Frequency Dropout do not operate on the sequence axis of speech-LLM encoder tokens.

## 3 Dropout and dropin in SALMONN

We describe our proposed two-phase training strategy that integrates dropout and dropin techniques into the SALMONN pipeline. The overall pipeline is illustrated in Figure 2. The primary LLM employed in our approach is SALMONN (Tang et al., 2023), an open-source model with a well-defined architecture: SALMONN leverages the Whisper (Radford et al., 2022) and the BEATs encoder (Chen et al., 2022) to extract and encode audio features into token sequences, enabling effective processing by LLMs. Additionally, Q-Former (Li et al., 2023) is used to project audio features conditioned on text instructions. After projecting audio and text features, they are further processed with LLaMA (Touvron et al., 2023) to generate the final outputs. In our two-stage training strategy, we adhere to this architecture while introducing Low-Rank Adaptation (LoRA) (Hu et al., 2021) to fine-tune Q-Former and the audio-text projection layers, keeping the other large components frozen to maintain computational efficiency.

**Figure 2:**
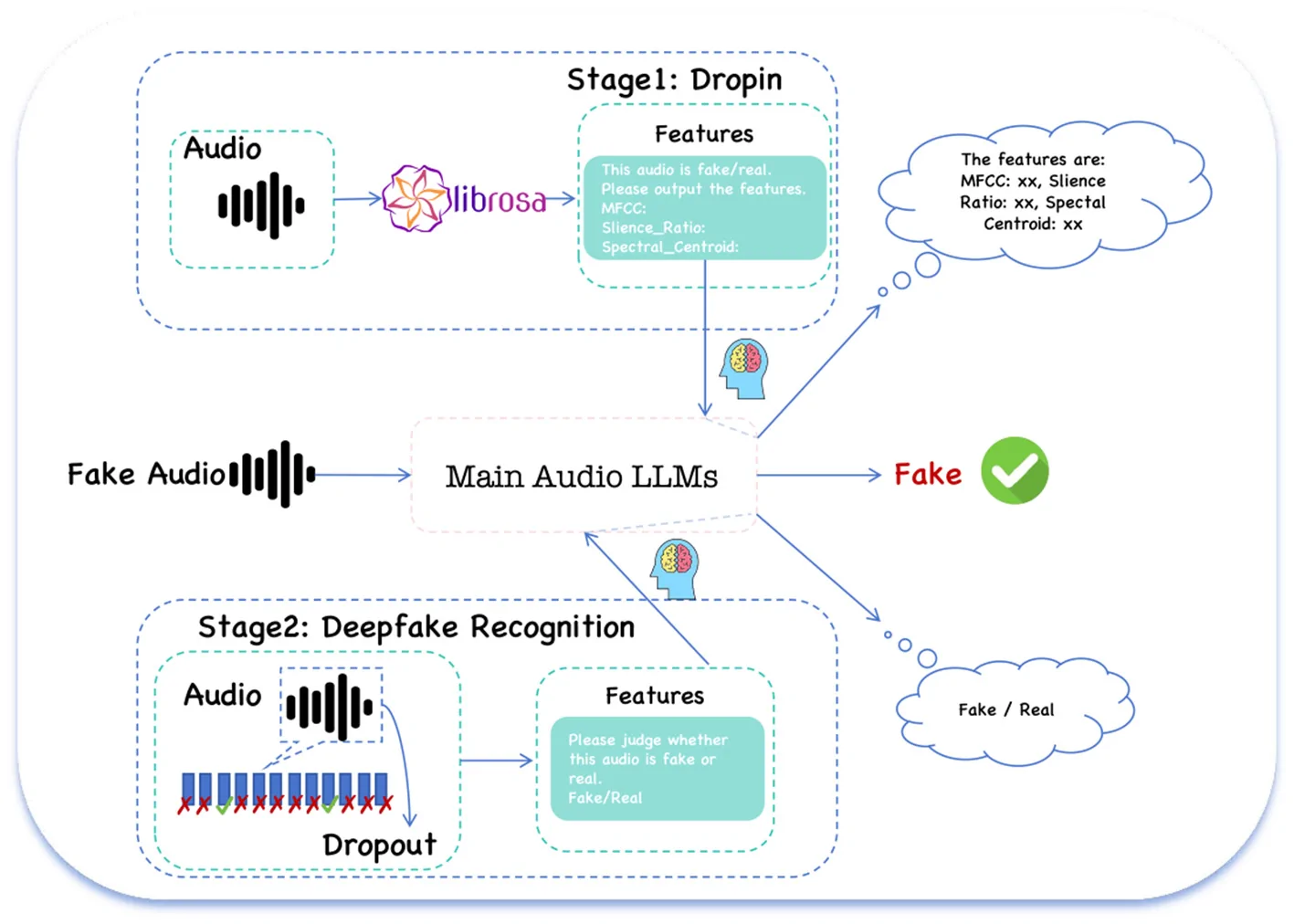
Two-stage training strategy with dropout and dropin for SALMONN. In Stage 1, the model is prompted with real or fake classifications and tasked with predicting key acoustic features, enhancing its feature comprehension. In Stage 2, randomly dropping out features forces the model to focus on meaningful representations before making the final classification.

### 3.1 Features dropin

We refer to Stage 1 as feature dropin, meaning dropping acoustic-feature knowledge into the LoRA adapters via an auxiliary regression pretext task, rather than physically inserting handcrafted feature values into the Stage 2 model input. Concretely, we fine-tune SALMONN's LoRA adapters to predict a small set of scalar acoustic descriptors (MFCC, SR, and SC) from the audio; the resulting adapter weights are then reused as the initialization for Stage 2, in which only the (sub-sampled) audio embedding and a neutral classification prompt are provided. More specifically, our goal is for the model to develop a deeper understanding of audio by learning the extracted acoustic features and linking them to classification decisions. To guide this learning process, we select Mel-Frequency Cepstral Coefficients (MFCC), Silence Ratio (SR), and Spectral Centroid (SC) as key features. Accordingly, we design the prompts as follows: “*This audio is* *y. Please output the features. MFCC: Silence Ratio: Spectral Centroid: ."*

We compute MFCC, SR, and SC using Librosa and treat them as ground-truth values *x*_*i*_. Specifically, the MFCC coefficients are first extracted from the audio signal, and then a mean over all frames and coefficients is used to obtain a single scalar value. We use a sampling rate of 16 kHz (resampled if needed), with other hyperparameter settings at their default values. The reason we aggregate frame-level representations by averaging across all frames is to reduce the output complexity of LLM predictions. Our preliminary smoke tests show that directly predicting fine-grained, frame-level values leads to unstable optimization and to failures at the dropout stage. Since the predicted features $\hat{x_{i}}$ are generated in natural language, we also represent the ground truth *x*_*i*_ in the same format. To train the model, we employ the Cross-Entropy Loss as the final loss function, enabling backpropagation through LoRA.

For each utterance we compute three ground-truth scalars, *x*_MFCC_, *x*_SR_, *x*_SC_, and render them as two-decimal strings *s*_*i*_ = round(*x*_*i*_, 2). The Stage-1 target sequence is the concatenation


$$\begin{array}{l} t=\text{\textasciitilde{}MFCC:\textasciitilde{}}s_{1}\text{\textasciitilde{}SR:\textasciitilde{}}s_{2}\text{\textasciitilde{}SC:\textasciitilde{}}s_{3}, \end{array}$$

tokenized by the SALMONN LLaMA tokenizer. Stage-1 optimizes token-level cross-entropy over this target string:


$$\begin{array}{l} L_{1}=-\frac{1}{\left|t\right|}\overset{\left|t\right|}{\underset{k=1}{\sum }}\log p_{\theta }\left(t_{k}|t_{<k},A,{\text{prompt}}_{1}\right), \end{array}$$ (1)

where θ denotes the LoRA parameters and *A* the input audio; gradients flow only through the LoRA adapters. Note that Equation 1 is a *string-level* cross-entropy, not a scalar regression loss; the LLM learns to emit the digits of *x*_*i*_ verbatim.

### 3.2 Dropout feature training

We introduce a windowed random retention operator over the SALMONN encoder token sequence. This is not the standard Bernoulli dropout (Srivastava et al., 2014); it is a deterministic-rate stochastic sub-sampler that partitions the sequence into non-overlapping windows and retains exactly one uniformly chosen token per window. Given that the SALMONN audio encoder produces high-dimensional embeddings and long sequence lengths, we apply a fixed-size windowing strategy to reduce dimensionality. Within the window, a single embedding index of length is randomly selected and retained, while the remaining embeddings are discarded, removing them from the feature sequence dimension. The window is then shifted to the right in a non-overlapping manner, excluding regions that have already been dropped—similar in spirit to a max pooling operation, shown in Figure 3. This process could be formulated as Equation 2:


$$\begin{array}{l} X_{\text{new}}=\left\{x_{i}|x_{i}\in X_{\text{ori}},i=rand\left(\text{windowsize}\right)\right\} \end{array}$$ (2)

**Figure 3:**
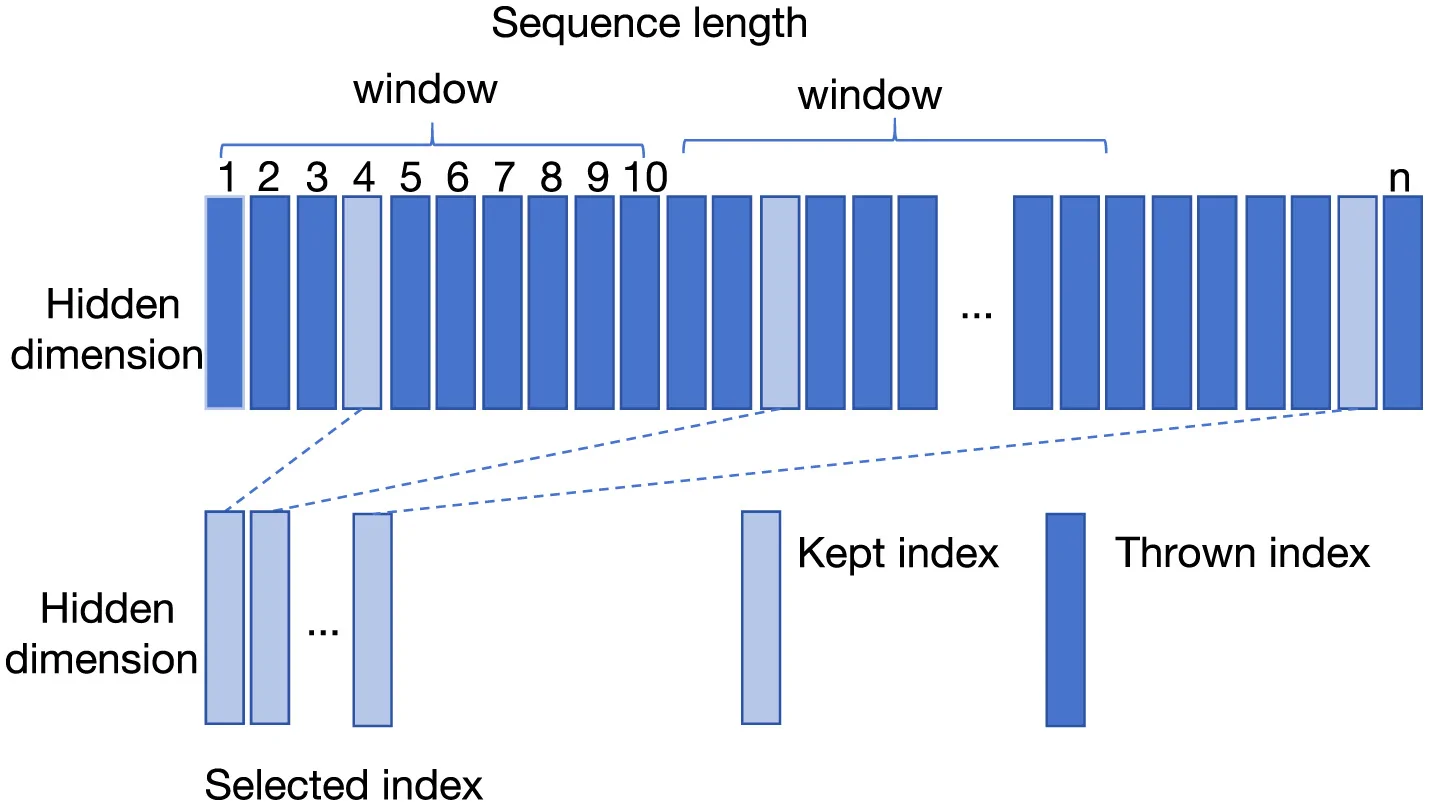
Dropout process. The randomly selected index of the sequence length is kept and recombined into a new sequence embedding. *N* is the original sequence length of the embedding obtained from the SALMONN audio encoder.

where *X*_new_ is the new feature embedding after original feature *X*_ori_ embedding dropout. This approach effectively reduces the number of active features—controlled by the window size—thereby minimizing interference in subsequent processing by the LLM. We use a window size of 10, which means we drop out 90% of the feature dimension. Notably, our dropout is applied to the feature embeddings rather than the conventional neural network dropout (Srivastava et al., 2014) or token-level dropout used in previous study.

### 3.3 Deepfake detection

The second stage, after applying dropout to the extracted features, focuses on decision-making. We feed the remaining features into the LLM using a structured prompt designed to guide classification after a full stage 1, the training of dropin. The model is then tasked with predicting the final label using the following prompt: “*Please judge whether this audio is fake or real."* The pseudocode is shown in Algorithm 1.

Algorithm 1 Deepfake detection with DropIn and DropOut.

**Input:** Audio sample *A*, original features *X*_ori_, window size *w*, pre-trained model *M* **Output:** Predicted label *y*
**Function DropOut(X**_ori_, ***w):***
Split window with window size *w*_*s*_ to get window slices
Randomly sample indices *i*~Uniform(1, |*X*_ori_|) within each window slice
Construct new feature subset: *X*_new_ = {*x*_*i*_∣*i*∈rand(*w*)}
**return** *X*_new_
**Function DropIn(A):**
Construct prompt and feed into LLM
**return** Trained features *F*_gen_
**Function DeepfakeDetect(A):** Extract original features *X*_ori_
*F*_gen_← DropIn(*A*)
*X*_new_← DropOut(*X*_ori_, *w*)
Construct new prompt *P* for detection
Compute prediction: *y* = *F*_gen_(*X*_new_, *P*)
**return** *y*

Stage-2 optimizes the token-level cross-entropy of the correct real/fake token as Equation 3:


$$\begin{array}{l} L_{2}=-\log p_{\theta }\left(y|X_{\text{new}},{\text{prompt}}_{2}\right),\;y\in \left\{\text{\textasciitilde{}real\textasciitilde{}},\text{\textasciitilde{}fake\textasciitilde{}}\right\}, \end{array}$$ (3)

where *X*_new_ is the encoder token sequence after feature dropout, and θ is initialized from the Stage-1 LoRA weights.

## 4 Experiments and analysis

### 4.1 Dataset

For our experiments, we select two widely used benchmark datasets: Fake or Real (FoR) (Reimao and Tzerpos, 2019) and ASVSpoof 2019 (Wang et al., 2020). We select the normalized subset in its entirety for FoR, as prior literature suggests it is both the most balanced and the most challenging. We retain the original training, validation, and test splits. For ASVSpoof 2019, we sample a representative portion for training due to GPU constraints for our experiments. The data list we use is in the Github repository: https://github.com/myxp-lyp/Defending-Against-Deepfake-Speech-Attacks-via-Feature-Dropout-and-Dropin-Regularization.git for reproduction. We have also selected M6 (Li et al., 2026) as our out-of-domain dataset. This dataset is designed for AI-generated music detection, a task similar to ours but one that represents a completely out-of-domain evaluation for our models. Specifically, we use the (f) subset, which consists of 1,000 real music samples and 500 AI-generated music samples.

We run the experiments on a Tesla V100-PCIE-32 GB, and the hyperparameters are in the code. These parameters follow the defaults from the original SALMONN repository. For settings that differ, we adjust them based on GPU memory. We have also run each experiment 5 times to validate the significance of the improvement in model performance. The number of training epochs is set to 30, based on preliminary experiments indicating that the model can effectively converge within a relatively small number of epochs. The hyperparameters are shown in Table 1.

**Table 1:** Hyperparameter settings.

Hyperparameter	Value
LoRA_alpha	32
LoRA_dropout	0.1
Max_text_len	300
Batch size	2
Early stop patience	5
Warmup learning rate	1 × 10^−6^
Epoch	30
Warmup steps	3,000
Start learning rate	3 × 10^−5^
Min learning rate	1 × 10^−5^
Weight decay	0.05
Beta2	0.999
Optimiser	Adam

### 4.2 Results

#### 4.2.1 Two stages vertical comparison on FoR

The results of our experiments are presented in Table 2, where we report two evaluation metrics: Equal Error Rate (EER) and Accuracy (Acc). These metrics are selected due to their widespread use in deepfake audio detection. In our evaluation, we derive a continuous score from the generative classifier using token log-probabilities. EER is then computed by sweeping a threshold over these scores and finding the operating point where the false acceptance rate equals the false rejection rate.

**Table 2:** Comparison of the effectiveness of the dropin and dropout methods with SALMONN on FoR data normalized subset.

Method	EER	Acc
TSSDNet (Hua et al., 2021)	0.375	0.645
Trident of Poseidon (Doan et al., 2024)	0.14	-
AASIST (Tak et al., 2022)	0.39	-
Statnet (Ranjan et al., 2022)	0.81	-
SEResNet34 (Dongre et al., 2022)	0.098	0.901
SALMONN	0.289_±0.011_	0.714_±0.019_
Dropout + SALMONN	0.177_±0.007, *p* < 0.01_	0.823_±0.013, *p* < 0.01_
Dropin + SALMONN	**0.096**_±0.004, *p* < 0.001_	**0.902**_±0.009, *p* < 0.001_
Dropout + Dropin + SALMONN	0.144_±0.005, *p* < 0.001_	0.857_±0.011, *p* < 0.001_

Best scores are marked in bold. A paired two-sided t-test assesses significance across five random seeds.

We visualize Mel-spectrograms in Figures 4–7, which show that the difference between real and fake audio is difficult to discern in the original spectrogram. However, while dropout is a random information-removal process, visualizing its effects reveals that fake audio spectrograms degrade differently from real ones, particularly at low and high frequencies. This suggests that fake audio may rely more heavily on fragile frequency components. Additionally, the time frames are different due to the dropout process. For visualization, encoder tokens that are retained after dropout are mapped back to their corresponding time regions in the input audio, and the spectrogram is displayed only for these selected segments. Consequently, the “dropout spectrograms” represent the time–frequency regions associated with the retained embeddings rather than a direct modification of the spectrogram itself.

**Figure 4:**
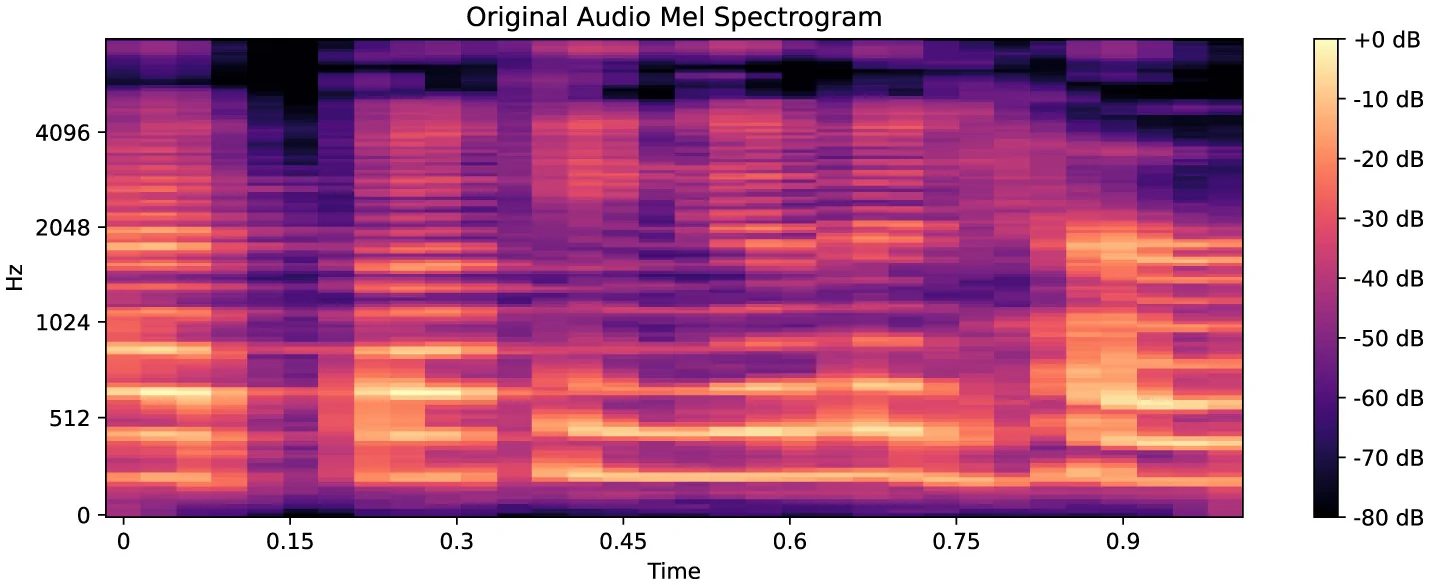
Original Mel spectrogram of real audio.

**Figure 5:**
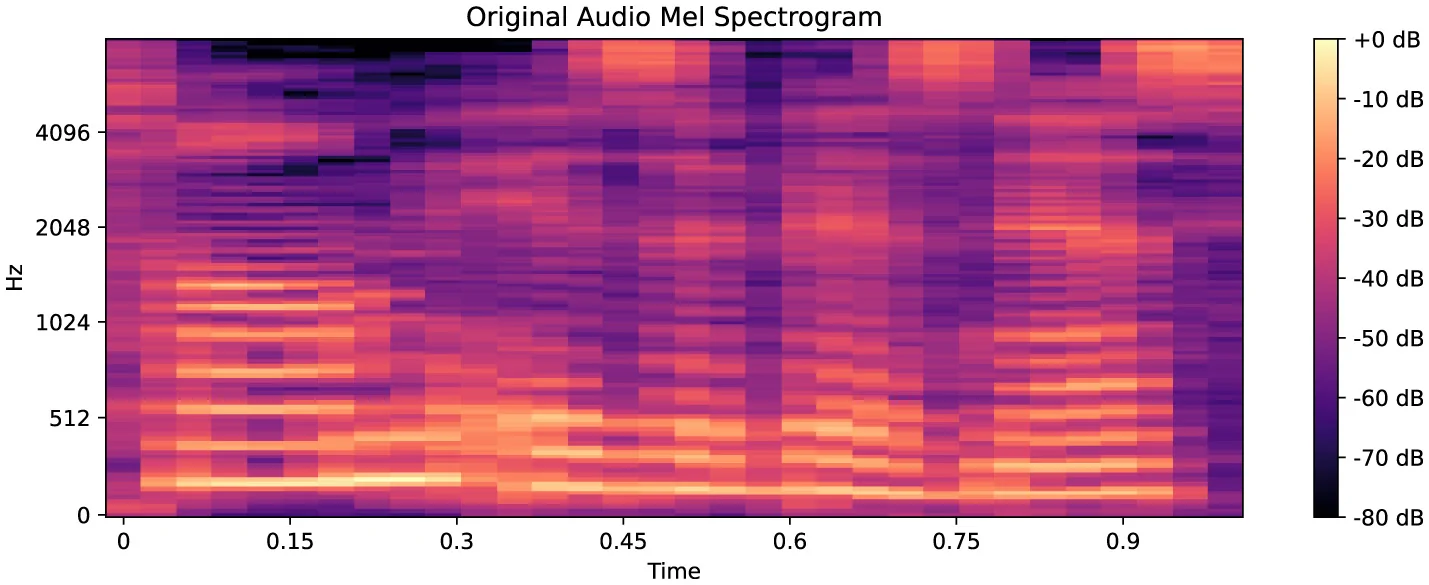
Original Mel spectrogram of fake audio.

**Figure 6:**
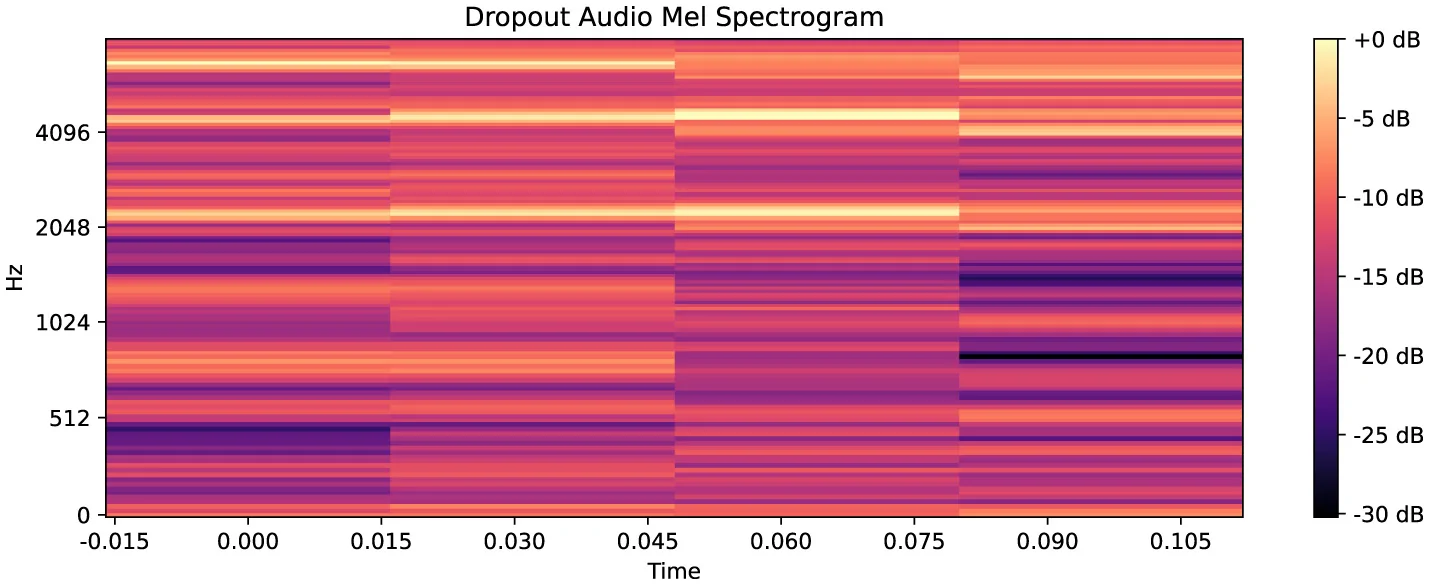
Dropout Mel spectrogram of real audio.

**Figure 7:**
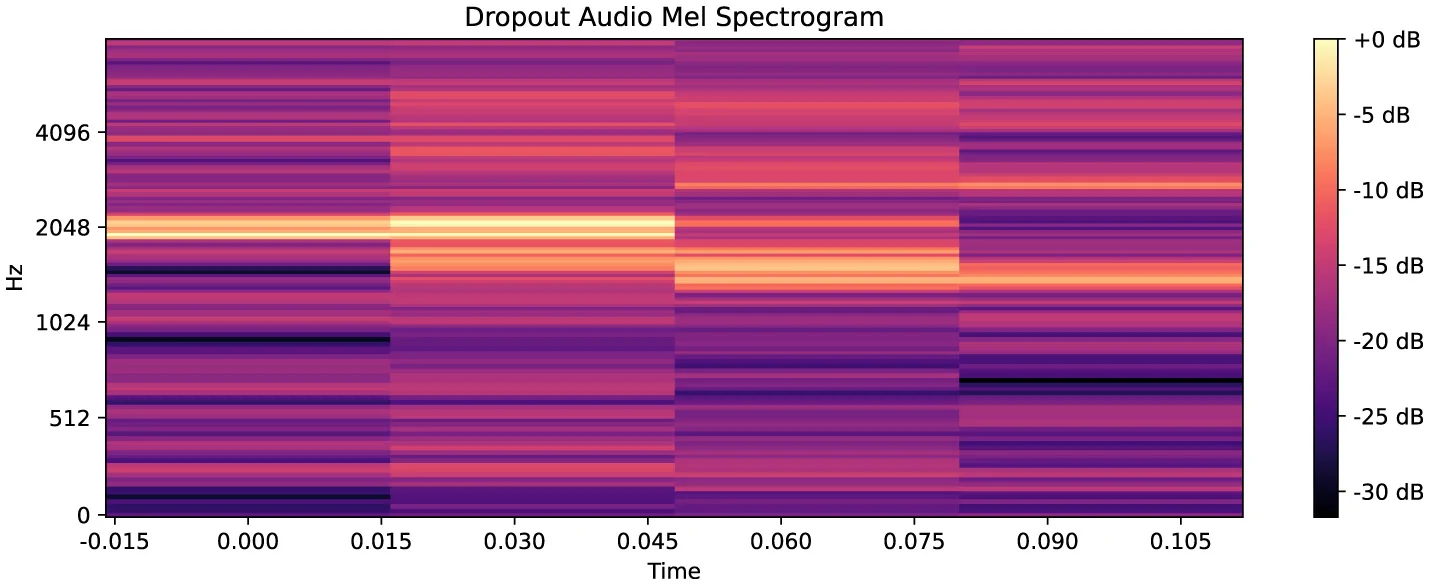
Dropout Mel spectrogram of fake audio.

From Table 2, the dropout and dropin strategies are effective in LLM training. Dropout, in particular, yields a considerable improvement of 0.109, and the dropin strategy greatly enhances model performance, with an absolute accuracy improvement of 0.188 and related EER improvements. These suggest that incorporating dropin features helps the model make more accurate predictions by leveraging additional, relevant information. The combination of the two strategies appears to yield slightly worse performance than using the dropin strategy alone, but better than the baseline. We believe this is because the dropin stage provides the model with greater capacity to learn by introducing additional features. However, when dropout is applied randomly, it may inadvertently eliminate features that could have contributed valuable information for the model to learn, potentially hindering the effectiveness of the dropin strategy. Our method also demonstrates strong performance, outperforming several existing baseline approaches.

#### 4.2.2 Horizontal comparison on ASVSpoof

We further strengthen the validity of our suggested approach on the ASVSpoof dataset. For this, we have sampled 5,160 samples for training, 5,096 for validation, and **full** 14,710 for testing from the ASVSpoof 2019 LA dataset, ensuring a 1:1 ratio of real to fake samples. These samples will be uploaded in the Github repository: https://github.com/myxp-lyp/Defending-Against-Deepfake-Speech-Attacks-via-Feature-Dropout-and-Dropin-Regularization.git. The results of different models are presented in Table 3. We adopt the tandem Detection Cost Function (t-DCF) as an evaluation metric in a more application-oriented context, as well as EER in percentage form. t-DCF is computed with the official ASVspoof 2019 evaluation package using its default cost parameters. We selected different metrics from FoR dataset for better comparison with other models in the literature. Unlike standalone metrics such as EER or accuracy, t-DCF jointly models the impact of spoofing countermeasures and automatic speaker verification (ASV) systems by simulating their combined deployment.

**Table 3:** Comparison of models on ASVspoof 2019 LA subset. Best scores are marked in bold.

Method	t-DCF	EER
CRNN-Spoof (Almutairi and Elgibreen, 2022)	0.132	0.043
SENet-34 (Almutairi and Elgibreen, 2022)	0.155	0.067
ResNet-34 (Almutairi and Elgibreen, 2022)	0.151	0.053
DBiLSTM (Almutairi and Elgibreen, 2022)	**0.008**	0.007
ConvNext-Tiny + Whisper (Pham et al., 2024)	-	0.030
Self attention (Huang and Pun, 2024)	0.104	0.045
SafeEar (Li X. et al., 2024)	0.149	0.031
WavLM (Pan et al., 2024)	-	0.007
AFP-Conformer (Huang et al., 2025)	0.045	0.016
FTC-SGAAMO (Lu et al., 2025)	0.031	0.010
SAM + Meta (Wang and Hansen, 2024)	0.028	0.005
SALMONN	0.0122	0.006_±0.0002_
Dropout + SALMONN	0.280	0.142_±0.0047, *p* < 0.01_
Dropin + SALMONN	0.010	**0.00496**_±0.0002, *p* < 0.001_
Dropin + Dropout + SALMONN	0.272	0.136_±0.0042, *p* < 0.001_

A paired two-sided *t*-test assesses significance across five random seeds.

Our model achieves the lowest Equal Error Rate (EER) of 0.00496, surpassing the performance of the ASVspoof 2019 models. Notably, it demonstrates an absolute EER reduction of 0.00244 compared to the best-performing existing model, highlighting the effectiveness of our proposed approach. Moreover, this is reached by sampling only about 20% of the training set, demonstrating the potential and efficiency of our dropin approach. This result highlights the utility of our proposed strategies.

We have also conducted an ablation study on the Dropout window size. The results show that increasing the window size generally degrades performance. A small window (*w* = 5 or 10) yields the lowest EERs (around 0.14%), while larger windows lead to a substantial increase in EER. This suggests that overly aggressive token sub-sampling can remove useful temporal information, thereby impairing detection performance, whereas a moderate window size can improve computational efficiency without substantially compromising performance.

However, interestingly, the dropout we introduced performs worse than the baseline on the ASVspoof dataset. We hypothesize that this is because ASVspoof contains subtle, distributed spoofing artifacts throughout the acoustic representation. Randomly dropping encoder tokens can inadvertently remove these informative cues, which are crucial for detecting spoofed audio. Nevertheless, introducing dropout remains a valid strategy to reduce model overfitting and cognitive burden, and it may still be beneficial in datasets where the relevant information is more robustly represented or less sparsely distributed. To ensure the validity of this finding, we directly evaluated the LLMs on the same test set without our training strategies. The results show 0.5 accuracy, suggesting that SALMONN has not encountered these test samples before and confirming the robustness of our approach.

#### 4.2.3 Out of domain test on M6

To evaluate the models trained on the aforementioned datasets with respect to their generalizability and robustness, given that prior research suggests the most challenging aspect lies in out-of-domain testing (Afchar et al., 2024). To assess this, we tested the best-performing model trained on FoR on an AI-generated music dataset, M6, as zero-shot testing. The results are presented in Table 4. On M6, our FoR-trained model reaches 0.677 accuracy, comparable to the ResNet18 baseline (0.685). We interpret this as evidence of transferable audio representations to a related but distinct domain

**Table 4:** Out-of-domain accuracy on the (f) subset of M6. We use the best-performing version of SALMONN for in-domain training.

Model	Accuracy
ResNet18 (P et al., 2020)	**0.685**
Vision-Transformer (Dosovitskiy et al., 2021)	0.630
Q-SVM (Cortes and Vapnik, 1995)	0.671
SALMONN (Ours)	0.677_±0.021, *p* < 0.05_

The other model results from SALMONN are obtained in (Li et al., 2024b). A paired two-sided t-test assesses significance across five random seeds. Bold means the best performing score.

#### 4.2.4 Ablation study of the audio projector

To investigate whether the audio projection layer is a crucial component of our suggested architecture, we employ Qwen2Audio (Chu et al., 2024), a state-of-the-art audio LLM that has demonstrated strong performance on various audio processing tasks, including automatic speech recognition (ASR). In our setup, we applied LoRA-based fine-tuning to the text-based core LLM of Qwen2Audio on the FoR dataset, while keeping the audio projector component from Whisper unchanged. However, the fine-tuned model consistently classified all audio inputs as fake. This outcome suggests that remapping audio features at the textual representation level is crucial for effective feature-level operations such as dropin and dropout.

#### 4.2.5 Robustness of our training strategy

As a final set of experiments, we evaluate our proposed dropin and dropout strategy for audio LLMs on the Listen, Think, and Understand (LTU) (Gong et al., 2023) task using the FoR dataset. LTU is an existing audio LLM designed for reasoning tasks, which we believe to have similar power in processing audio data. We employ LoRA for fine-tuning, using the same hyperparameters as SALMONN but with fewer training epochs due to LTU's smaller parameter count. The results, including accuracy, recall, precision, and F1, are listed in Table 5.

**Table 5:** Comparison of the effectiveness of the dropin and dropout methods with LTU on the FoR data normalized subset.

Method	Acc	Recall	Precision	F1
LTU	0.511_±0.018_	0_±0.000_	0_±0.000_	-
Dropout + LTU	0.512_±0.017, *p*>0.05_	0.103_±0.012, *p*>0.05_	0.501_±0.021, *p*>0.05_	0.171_±0.015, *p*>0.05_
Dropin + LTU	0.524_±0.014, *p* < 0.01_	0.501_±0.022, *p* < 0.01_	**0.996**_±0.004, *p* < 0.001_	0.666_±0.013, *p* < 0.01_
Dropout + Dropin + LTU	**0.571**_±0.010, *p* < 0.001_	**0.923**_±0.006, *p* < 0.001_	0.535_±0.018, *p* < 0.01_	**0.678**_±0.011, *p* < 0.001_

Best scores are marked in bold.

The results show the generalizability of Dropin and Dropout strategies across various settings and LLMs. It is shown that the baseline LTU model exhibits poor performance, with an accuracy of 0.511 and a precision of 0, as it predicts all samples as fake. Introducing Dropout alone slightly improves the model's accuracy to 0.512, indicating that the model is no longer producing the same prediction for every sample.

Notably, incorporating Dropin alone considerably boosts both recall to 0.501 and precision to 0.996, resulting in an overall accuracy of 0.524, highlighting Dropin's ability to augment the model's sensitivity to informative instances. When Dropin and Dropout are combined on LTU/FoR, the model achieves its best performance in this setting (Acc 0.571, Recall 0.923). Given the opposite trend on ASVspoof 2019 LA, we describe this complementarity as model- and dataset-specific rather than general.

## 5 Conclusion

We propose a two-stage training strategy for speech LLMs in deepfake detection. First, the model is trained to predict key audio features from labeled examples, enhancing audio sensitivity. Second, feature-level dropout reduces input dimensionality and improves robustness. Our model achieves strong results on the FoR dataset, attains competitive performance on ASVSpoof 2019 LA, and generalizes to AI-generated music detection. However, our strategy relies on the base audio LLMs. Training an audio LLM from scratch will be extremely expensive. Also, the out-of-domain deepfake detection accuracy still has room for improvement. Future study may include further analysis of the impact of selected dropin features on model performance, as well as additional acoustic features to be dropped, such as prosody or phase information. Several directions remain open, such as broader evaluation on unseen benchmarks, validation on robustness to newer generative attacks, improving computational efficiency and inference cost, and providing interpretability of the selected acoustic features.

## Data Availability

Publicly available datasets were analyzed in this study. This data can be found here: ASVSpoof2019.
